# Mechanisms of initiation of cortical spreading depression

**DOI:** 10.1186/s10194-023-01643-9

**Published:** 2023-08-08

**Authors:** Marina Vitale, Angelita Tottene, Maral Zarin Zadeh, KC Brennan, Daniela Pietrobon

**Affiliations:** 1 Department of Biomedical Sciences, University of Padova, 35131 Padova, Italy; 2 Department of Neurology, University of Utah School of Medicine, UT 84108 Salt Lake City, USA; 3 Padova Neuroscience Center (PNC), University of Padova, 35131 Padova, Italy

**Keywords:** Migraine, Cortical spreading depression, Spreading depolarization, Cerebral cortex, Glutamate NMDA receptors, Voltage-gated calcium channels

## Abstract

**Background:**

There is increasing evidence from human and animal studies that cortical spreading depression (CSD) is the neurophysiological correlate of migraine aura and a trigger of migraine pain mechanisms. The mechanisms of initiation of CSD in the brain of migraineurs remain unknown, and the mechanisms of initiation of experimentally induced CSD in normally metabolizing brain tissue remain incompletely understood and controversial. Here, we investigated the mechanisms of CSD initiation by focal application of KCl in mouse cerebral cortex slices.

**Methods:**

High KCl puffs of increasing duration up to the threshold duration eliciting a CSD were applied on layer 2/3 whilst the membrane potential of a pyramidal neuron located very close to the site of KCl application and the intrinsic optic signal were simultaneously recorded. This was done before and after the application of a specific blocker of either NMDA or AMPA glutamate receptors (NMDARs, AMPARs) or voltage-gated Ca^2+^ (Ca_V_) channels. If the drug blocked CSD, stimuli up to 12–15 times the threshold were applied.

**Results:**

Blocking either NMDARs with MK-801 or Ca_V_ channels with Ni^2+^ completely inhibited CSD initiation by both CSD threshold and largely suprathreshold KCl stimuli. Inhibiting AMPARs with NBQX was without effect on the CSD threshold and velocity. Analysis of the CSD subthreshold and threshold neuronal depolarizations in control conditions and in the presence of MK-801 or Ni^2+^ revealed that the mechanism underlying ignition of CSD by a threshold stimulus (and not by a just subthreshold stimulus) is the Ca_V_-dependent activation of a threshold level of NMDARs (and/or of channels whose opening depends on the latter). The delay of several seconds with which this occurs underlies the delay of CSD initiation relative to the rapid neuronal depolarization produced by KCl.

**Conclusions:**

Both NMDARs and Ca_V_ channels are necessary for CSD initiation, which is not determined by the extracellular K^+^ or neuronal depolarization levels per se, but requires the Ca_V_-dependent activation of a threshold level of NMDARs. This occurs with a delay of several seconds relative to the rapid depolarization produced by the KCl stimulus. Our data give insights into potential mechanisms of CSD initiation in migraine.

## Background

Cortical spreading depression (CSD) is a slowly propagating self-sustaining wave of nearly complete depolarization of a sizable population of brain cells that lasts about one minute and silences brain electrical activity for several minutes (hence the name spreading depression) [1–3]. There is increasing evidence from human and animal studies that CSD is the neurophysiological correlate of migraine aura and a trigger of the migraine pain mechanisms ( [3–5] and references therein; [6–8]). A key unanswered question in migraine neurobiology concerns the mechanisms that make the brain of migraineurs susceptible to CSD. Important insights into this question can be obtained by studying the molecular and cellular mechanisms of initiation of the “experimental” CSD, which can be induced in normally metabolizing brain tissue by intense depolarizing stimuli [2, 3]. Despite recent progress, these mechanisms remain incompletely understood and controversial. This is largely due to the fact that CSD is a complex phenomenon characterized by a sequence of different phases involving different mechanisms and different channels each yielding the necessary condition for the next to open, and this makes the interpretation of the available (mainly pharmacological) data difficult [3].

In vivo measurements of [K]_e_ [9] and, only recently, extracellular glutamate [10] at the CSD initiation site in response to CSD subthreshold and threshold depolarizing stimuli support the idea that increases of [K]_e_ and/or extracellular glutamate above critical threshold values are key initiating events for CSD ignition. The idea that an uncontrolled rise of [K]_e_ and of spilled glutamate can cooperate in the generation of CSD is also supported by modeling studies aimed to define the minimal biophysical machinery capable of initiating CSD [2, 11]. According to these studies, the generation of a net self-sustaining inward current across the dendritic membrane is necessary to initiate the positive feedback cycle that makes the initial gradual neuronal depolarization self-regenerative and confers to CSD its all-or-none characteristics [2, 11].

However, the nature of the ion channels whose activation by depolarizing CSD-inducing stimuli is necessary for (or involved in) the generation of the net self-sustaining inward current that initiates the CSD positive feedback cycle remains incompletely understood. The glutamate NMDA receptors (NMDARs) appear as optimal candidates, given that their activation depends on both glutamate and membrane depolarization (and hence [K]_e_) and that they are highly expressed in the apical dendrites of pyramidal cells, which appear preferentially involved in CSD initiation [2]. However, even limiting the discussion to the mechanisms of CSD induced by focal electrical and/or high KCl stimuli, the role of NMDARs in CSD initiation remains unclear and controversial [3, 12, 13]. The involvement of NMDARs in CSD initiation appears to be clearly supported by the higher CSD stimulation threshold measured after partial inhibition of NMDARs [14], which stands in contrast with recent claims of lack of involvement of NMDARs in CSD initiation [12]. The consistent findings that blocking the NMDARs completely inhibits CSD measured far (≥ 500 μm) from the site of focal stimulation in the cerebral cortex (even when largely suprathreshold stimuli are used to induce it both in vivo and in vitro) [14–16] do not allow to distinguish whether NMDARs are necessary for CSD initiation or CSD propagation (or both). But there is independent evidence that NMDARs are necessary for CSD propagation (e.g. [17].). The few studies in which CSD was measured close to the site of focal stimulation in the presence of NMDAR antagonists report conflicting findings [3]. Thus, whether NMDARs are necessary for CSD initiation remains unsettled.

Considering the source of glutamate for the activation of NMDARs, there is clear support for the involvement of the neuronal voltage-gated Ca_V_2.1 calcium channels (the Ca_V_ channels which play a dominant role in controlling glutamate release [18–20]) in CSD initiation by focal electrical or high KCl stimulation. In fact, a lower CSD stimulation threshold was measured in familial hemiplegic migraine type 1 (FHM1) knockin mice carrying a gain-of-function mutation in the Ca_V_2.1 channel [21, 22], and a causative link was established between the enhanced glutamate release at cortical synapses produced by the FHM1 mutation and the facilitation of CSD initiation [23]. On the other hand, mutant mice carrying mutations, which cause partial loss-of-function of Ca_V_2.1 channels and reduced K^+^-evoked glutamate rise, showed an increased CSD stimulation threshold [24]. Moreover, complete inhibition of Ca_V_2.1 channels completely inhibited CSD measured *far* from the focal stimulation site even when largely suprathreshold stimuli were applied [16]. Although these findings support the involvement of Ca_V_2.1 channels and/or Ca_V_2.1-dependent glutamate release in CSD initiation, they do not allow one to draw conclusions regarding their necessity for CSD initiation.

Here, we investigated the mechanisms of CSD initiation by focal application of high KCl in mouse cerebral cortex acute slices. To do this, we simultaneously recorded the membrane potential of layer 2/3 (L2/3) pyramidal neurons located very close to the site of KCl application and the intrinsic optic signal (IOS) at this site in response to depolarizing stimuli of increasing intensity (up to the threshold stimulus eliciting a CSD in control and up to a largely suprathreshold stimulus after perfusion of saturating NMDARs or Ca_V_ channels blockers). We show that the mechanism underlying the ignition of CSD by a threshold stimulus and not by a just subthreshold stimulus is the Ca_V_-dependent activation of a threshold level of NMDARs (and/or of channels whose opening depends on the latter) and that the delay with which this occurs underlies the delay of CSD initiation relative to the rapid neuronal depolarization produced by the KCl stimulus. Moreover, we show that both NMDARs and voltage-gated calcium channels are necessary for CSD initiation.

## Methods

### Animals

Experiments were performed using wild-type C57BL6J mice or GIN mice, obtained by crossbreeding C57BL6J mice with homozygous FVB-Tg(GadGFP)45704Swn/J (GIN) mice expressing GFP in a subset of somatostatin-expressing interneurons [25, 26]. Animals were housed in specific pathogen free conditions, maintained on a 12-h light/dark cycle, with free access to food and water. All experimental procedures involving animals and their care were carried out in accordance with Italian laws and policies (D.L. n. 26, March 14, 2014) and with the guidelines established by the European Community Council Directive (2010/63/UE) and were approved by the local authority veterinary services in Padova (Italy Aut. Min. 652/2015-PR and 340/2022-PR).

### Acute brain slice preparation

Acute coronal slices containing the somatosensory barrel cortex were prepared from postnatal day P 17–20 male and female mice, as described in [27]. Briefly, animals were anesthetized with isoflurane and decapitated. The brain was quickly removed and put in an ice-cold cutting solution (in mM: 130 K gluconate, 15 KCl, 0.2 EGTA, 20 HEPES, 25 glucose, 2 kynurenic acid, 5 × 10^− 5^ minocycline, pH 7.4 with NaOH, oxygenated with 100% O_2_) [28]. 350 μm-thick slices were then cut on the coronal plane with a vibratome (VT1200S, Leica Biosystems, Germany) and were transferred for 1 min in a solution containing (in mM) 225 D-mannitol, 2.5 KCl, 1.25 NaH_2_PO_4_, 26 NaHCO_3_, 25 glucose, 0.8 CaCl_2_, 8 MgCl_2_, 2 kynurenic acid, 5 × 10^− 5^ minocycline, saturated with 95% O_2_ and 5% CO_2_. Slices were then maintained at 30 °C for 30 min in standard artificial cerebrospinal fluid saturated with 95% O_2_ and 5% CO_2_ (sACSF in mM: 125 NaCl, 2.5 KCl, 25 NaHCO_3_, 1.25 NaH_2_PO_4_, 1 MgCl_2_, 2 CaCl_2_, 25 glucose) plus 50 nM minocycline, and then transferred at room temperature in the same solution for a minimum of 30 min before being used for the experiment. All experiments were performed within 6 h from the mouse decapitation.

### Patch-clamp recordings

Whole-cell patch-clamp recordings were made following standard techniques. Electrical signals were recorded through a Multiclamp 700B amplifier and digitized using an Axon Digidata 1550 interface and pClamp software (Molecular Devices). Pipette resistance: 3–4 MΩ.

Brain slices were continuously perfused in a submersion chamber with a fresh extracellular solution at room temperature at a flow rate of 3 ml/min using a peristaltic pump (Miniplus 3, Gilson). The extracellular solution contained: 125 mM NaCl, 3.5 mM KCl, 25 mM NaHCO_3_, 1.25 mM NaH_2_PO_4_, 0.5 mM MgCl_2_, 1 mM CaCl_2_, 25 mM glucose (saturated with 95% O_2_ and 5% CO_2_). Membrane potential recordings were made from upper L2/3 pyramidal cells deeper than 45 μm from the slice surface. The cells were visualized using an upright microscope (Nikon Eclipse; Nikon, Tokyo, Japan) equipped with infrared light and infrared differential interference contrast optics (water-immersion objective 60×) and identified by their typical morphological pyramidal shape and the presence of a prominent apical dendrite and their spiking pattern in response to 600 ms pulses of depolarizing current of increasing intensity [23]. The slices were used for recording only if more than 50% of the cells were alive at 45 μm depth in a 228 × 172 μm field.

The internal solution contained: 114 mM K-gluconate, 6 mM KCl, 4 mM MgATP, 0.3 mM NaGTP, 10 mM Na-phosphocreatine, 10 mM HEPES, 30 mM sucrose (pH = 7.25 with KOH).

### Cortical spreading depression

**Fig. 1:**
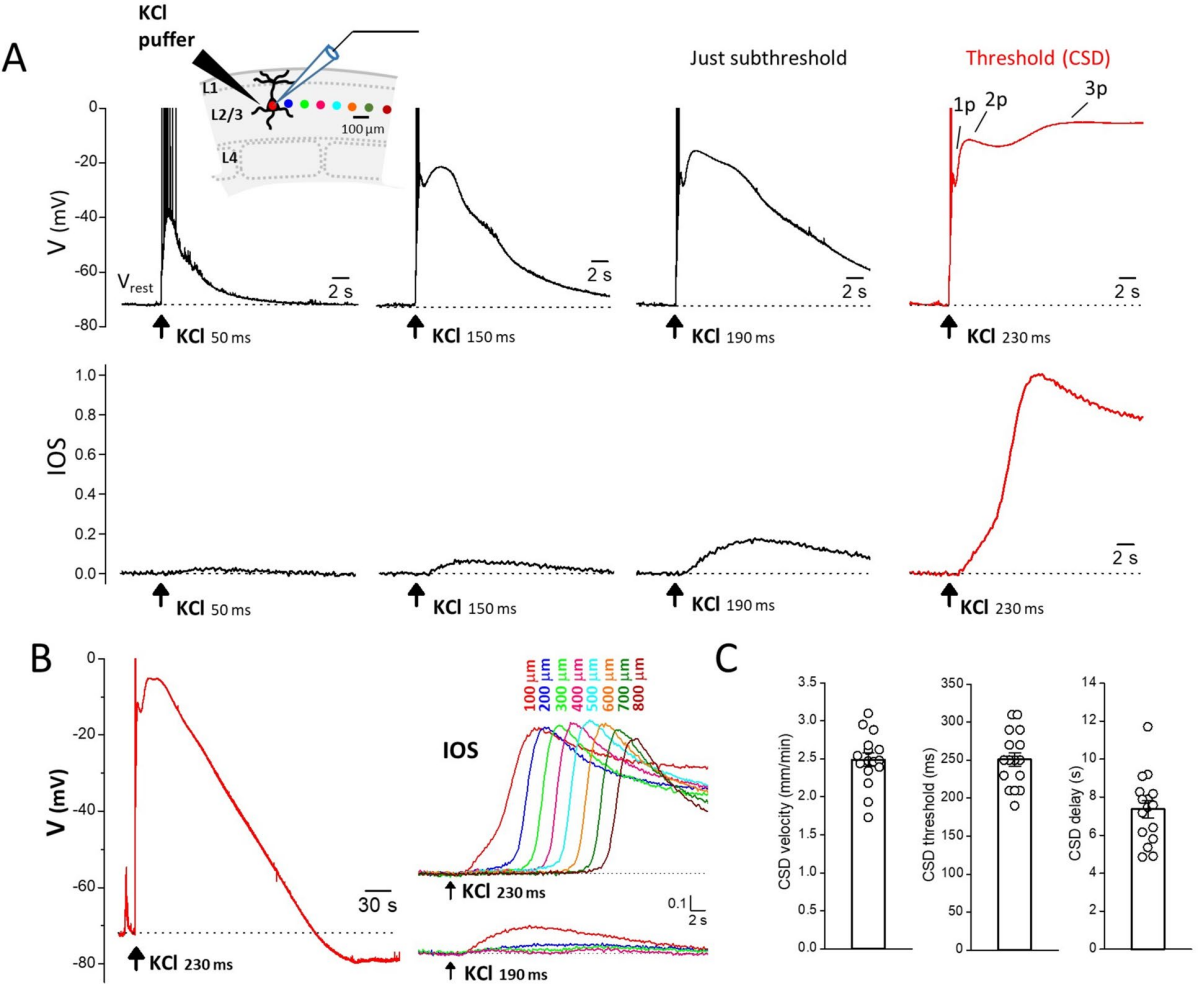
Neuronal voltage changes and IOS changes near the site of CSD induction elicited by CSD subthreshold and threshold KCl stimuli. **A** High KCl puffs of increasing duration were applied onto the slice surface (L2/3) up to the threshold duration eliciting a CSD and, simultaneously, the IOS at the site of KCl application and at different distances from it (indicated by the colored dots in the inset) and the membrane potential of a L2/3 pyramidal neuron located at 100 µm from the KCl puffer were recorded (inset). Upper panels: representative membrane potential changes recorded in a L2/3 pyramidal cell located at 100 µm from the site of application of KCl puffs of increasing duration (as indicated below the black traces) up to the threshold duration eliciting a CSD (red trace). The voltage values of the first two peaks of the depolarization produced by the just subthreshold stimulus (-25 and -16 mV, respectively) are similar to those of the first two peaks of the depolarization produced by the CSD threshold stimulus (indicated with 1p and 2p in the threshold trace: -25 and -12 mV, respectively). The CSD threshold stimulus produces a third depolarization peak to -5 mV. Lower panels: normalized IOS changes recorded simultaneously at the same site in response to the same KCl puffs; the traces were normalized relative to the amplitude of the IOS change induced by the CSD threshold stimulus. The beginning of the steep IOS rise produced by the threshold KCl stimulus (red trace) occurs with a delay of 6.6 s from the KCl application and temporally coincides with the development of the third peak of the voltage change produced by the same stimulus. **B** Left: entire membrane potential change comprising the repolarization phase following the peak of the depolarization elicited by the CSD threshold KCl stimulus (same CSD trace as in panel A; duration at half amplitude, HW = 123 s). Right: IOS changes recorded at different distances from the KCl puffer (as indicated above the traces and cf inset in panel A for color code) in response to the CSD threshold (upper panel) and just subthreshold (lower panel) KCl stimulus in the same representative experiment. The steep IOS rise elicited by the CSD threshold stimulus propagates at a rate of 2.9 mm/min. **C** Left: average rate of propagation of CSD: 2.49 ± 0.09 mm/min; average CSD threshold: 251 ± 9 ms; average delay of CSD relative to the KCl puff: 7.4 ± 0.5 s (n = 16, *N* = 15)

CSD was elicited in acute cortical coronal slices as in [23]. Briefly, the brain slices were placed into a submersion chamber and continuously perfused with fresh extracellular solution at room temperature at a flow rate of 3 ml/min. Brief pressure-ejection pulses of 3 M KCl (0.5 bar) of increasing duration (at 5 or 8 min intervals) were applied through a glass micropipette (resistance ranging from 0.20 to 0.23 MΩ) onto the slice surface on L2/3, using a PDES-02DX pneumatic drug ejection system (Npi Electronic GmbH, Tamm, Germany), until a CSD was elicited. For each KCl stimulus of increasing duration, the IOS changes at the tip of the KCl puffer and at increasing distances from it and the membrane potential of a L2/3 pyramidal cell located at 100 μm from the tip of the KCl puffer were simultaneously recorded. CSD was detected by the typical long-lasting depolarization to almost 0 mV in the patch-clamped L2/3 pyramidal cell and/or by the typical propagating steep change in IOS (Fig. 1). The duration of the first pulse eliciting a CSD was taken as CSD threshold and the rate of horizontal spread of the change in IOS as CSD velocity. IOS was recorded using a CMOS camera (Basler ace acA1920-155 μm USB 3.0, Basler, Germany) connected to the upright microscope (Nikon Eclipse; 10× magnification). Images were recorded at 200 ms intervals as 1920 × 1200 pixels images (pixel size: 1.27 μm). MBF ImageJ software was used for the offline analysis of the digitalized images. The IOS change is expressed as change in light transmittance relative to the background signal.

The effect of blocking different types of channels on the depolarization and the IOS elicited by CSD subthreshold, threshold or slightly suprathreshold KCl stimuli was investigated by first measuring the control CSD threshold as described above, and then, after 30 min, measuring the depolarization and the IOS change induced by the same KCl stimuli in the presence of a saturating concentration of a specific channel blocker which had been perfused for 15–25 min (as specified in Results). A CSD-inducing KCl stimulus was considered a threshold stimulus if the largest subthreshold KCl stimulus applied during the measurement of the control CSD had a duration only 20–40 ms lower than the threshold stimulus (i.e. it was just subthreshold). A CSD-inducing KCl stimulus was considered a slightly suprathreshold stimulus if the largest subthreshold KCl stimulus applied during the measurement of the control CSD had a duration more than 40 ms lower than the threshold stimulus. The effect of blocking different types of channels on the depolarization and the IOS elicited by largely suprathreshold KCl stimuli was investigated by first measuring the control CSD threshold as described above, and then, after 30 min, measuring the depolarization and the IOS change induced by KCl stimuli of duration up to 12–15 times longer than the CSD threshold (or slightly suprathreshold) stimulus in the presence of a saturating concentration of a specific channel blocker, which had been perfused for 15–25 min (as specified in Results).

Control IOS experiments were performed to verify that after 30 min from the induction of the CSD in control conditions the post-CSD refractory period has ended and there are no other relevant post-CSD effects. Indeed, the CSD threshold remained unaltered after 30 min of sham (extracellular solution) perfusion following the assessment of the control CSD threshold (264 ± 16 ms sham vs. 259 ± 17 ms control, *n* = 7, *N* = 2, Wilcoxon signed rank test, *p* = 0.8) and the CSD velocity was only slightly reduced (2.43 ± 0.06 mm/min sham vs. 2.72 ± 0.05 mm/min control, *n* = 7, *N* = 2, paired t-test, *p* = 0.0002).

Series resistance was monitored throughout the experiment; experiments with series resistance > 30 MΩ were excluded from the data. The fact that often the series resistance increased and/or the seal deteriorated after the induction of the control CSD limited the numerosity of the experiments in which we could record the membrane potential of the pyramidal cell before and after drug perfusion in response to both subthreshold and threshold stimulation.

The CSD threshold and velocity measured in C57BL6J mice and GIN mice (with 75% C57BL6J genetic background) were similar (CSD threshold: 251 ± 12 ms, *n* = 14, *N* = 13 vs. 247 ± 10 ms, *n* = 19, *N* = 12, MW test *p* = 0.8; CSD velocity: 2.32 ± 0.11 mm/min, *n* = 14, *N* = 13 vs. 2.54 ± 0.07, *n* = 19, *N* = 12, t-test *p* = 0.09). Hence the data were pooled.

### Statistics

Statistical analyses were performed with Statgraphics centurion XVII software (RRID: SCR_015248). After assessing for normal distribution (using the Shapiro–Wilk normality test), comparison between two groups was made using two-tailed unpaired or paired *t* test for normally distributed data and the Mann–Whitney (MW) or Wilcoxon signed rank tests for nonparametric data. Equal variances were assumed. Data are given in the text and figures as mean ± SEM. The significance level was set at *p* < 0.05 (*P < 0.05; **P < 0.01; ***P < 0.001).

The number *n* of observations (reported in the text and legends to figures) indicates the number of cells or slices recorded from, and the number *N* indicates the number of mice from which the data were obtained. No statistical methods were used to choose sample sizes that were estimated based on previous experience and are in line with those in the literature.

## Results

To investigate the mechanisms of initiation of CSD by focal application of high KCl to acute cortical slices, we applied high KCl puffs of increasing duration (i.e. depolarizing stimuli of increasing intensity) onto the slice surface (L2/3) up to the threshold duration eliciting a CSD, and simultaneously recorded the IOS at the site of KCl application and the membrane potential of L2/3 pyramidal neurons located very close to this site (at 100 μm from the KCl puffer) (Fig. 1A, inset). The representative experiment in Fig. 1 shows that the CSD subthreshold KCl stimuli produce neuronal depolarizations which increase and change shape with increasing puff duration, being characterized, at low KCl, by a single peak and, at higher KCl, by a second peak after the initial rapid depolarization. With KCl stimuli approaching the CSD threshold, a hint of a third peak becomes evident as a shoulder following the second peak and/or as a prolongation of the overall depolarization (Fig. 1A, black traces in upper panel). A burst of action potentials was usually present in the rising phase of the first peak depolarization (and on top of the single peak depolarization at low KCl). The CSD threshold KCl stimulus induced the typical CSD depolarization (Fig. 1A, B red traces), characterized by a third peak to almost 0 mV (-3.4 ± 0.7 mV, *n* = 16, *N* = 15) following the first two peaks and by a much longer duration than the just subthreshold depolarization (116 ± 4 s vs. 11.7 ± 0.5 s duration at half amplitude, HW; paired t-test: *p* = 2 × 10^− 13^). However, the membrane potential values of the first peak of the depolarization induced by CSD threshold and just subthreshold (20–40 ms smaller puff duration than the threshold) stimuli were similar (-18.7 ± 1.2 mV and − 19.2 ± 1.2 mV, respectively, *n* = 16, *N* = 15, paired t-test: *p* = 0.09), and the membrane potential value of the second peak of the depolarization induced by CSD threshold stimuli was only slightly higher than that induced by just subthreshold stimuli (-14.9 ± 1.3 mV vs. -18.6 ± 1.1 mV, *n* = 16, *N* = 15, paired t-test: *p* = 0.000015).

The lower panels in Fig. 1A show the IOS changes measured at the location of the recorded pyramidal cell. The CSD threshold KCl stimulus induced the steep large IOS change typical of CSD (Fig. 1A, red trace in the lower panel), whose amplitude did not decrease with distance and propagated at an average rate of 2.49 ± 0.09 mm/min (*n* = 16, *N* = 15) (Fig. 1B, C). In contrast, the subthreshold KCl stimuli produced small, slow IOS changes (Fig. 1A, black traces in lower panel), which did not propagate; their amplitude declined very rapidly with increasing distance from the KCl puffer, being close to zero at 200 μm (as shown in Fig. 1B for the just subthreshold stimulus*).* The steep IOS rise typical of CSD began with a certain delay following a slower, smaller IOS rise, which did not propagate, and whose amplitude rapidly declined with distance from the KCl puffer (as the amplitude of the slow rise produced by the just subthreshold depolarization) (Fig. 1A, B). The beginning of CSD, as obtained from the beginning of the steep IOS rise, occurs with a delay of several seconds relative to the time of KCl application (7.4 ± 0.5 s, at the location of the recorded pyramidal cell; *n* = 16; *N* = 15; Fig. 1C). Comparison with the simultaneously recorded neuronal membrane potential shows that the beginning of CSD does not coincide with the nearly immediate neuronal depolarization produced by the CSD threshold KCl puff. It occurs after the first two depolarization peaks and appears to temporally overlap with the development of the third depolarization peak (Fig. 1A).

What is the mechanism underlying the slow development of the CSD depolarization and its delay relative to the rapid depolarization produced by the CSD threshold KCl stimulus? And what is the mechanism underlying the ignition of CSD by a threshold stimulus and not by a just subthreshold stimulus which produces a rapid depolarization only slightly smaller than that at the threshold? It is very unlikely that it is the amplitude of the early depolarization per se that determines the ignition of CSD because at threshold it was quite variable in different experiments and there was a large overlap with the values of the early depolarization at just subthreshold (the voltage values of second peak varied from − 26 to -7 mV at threshold and from − 27 to -11 mV at just subthreshold in *n* = 16 experiments; moreover, see Figs. 4 and 6). The changes in amplitude and shape of the depolarizations produced by the subthreshold stimuli (Fig. 1A) suggest that the sequential activation of different cationic channels, opening with a different time course and a different dependence on the K^+^ stimuli, may underlie the development of the second peak and of the later shoulder (and/or prolongation of the depolarization) with increasing stimulus intensity. To test the hypothesis that the level of activation of one (or more) of these channels is critical for CSD ignition and to investigate in particular the role of NMDARs, we studied the effect of the NMDAR antagonist MK-801 on the depolarization elicited by CSD threshold and subthreshold KCl stimuli in pyramidal cells located at the site of CSD induction (at 100 μm from the KCl puffer).

**Fig. 2:**
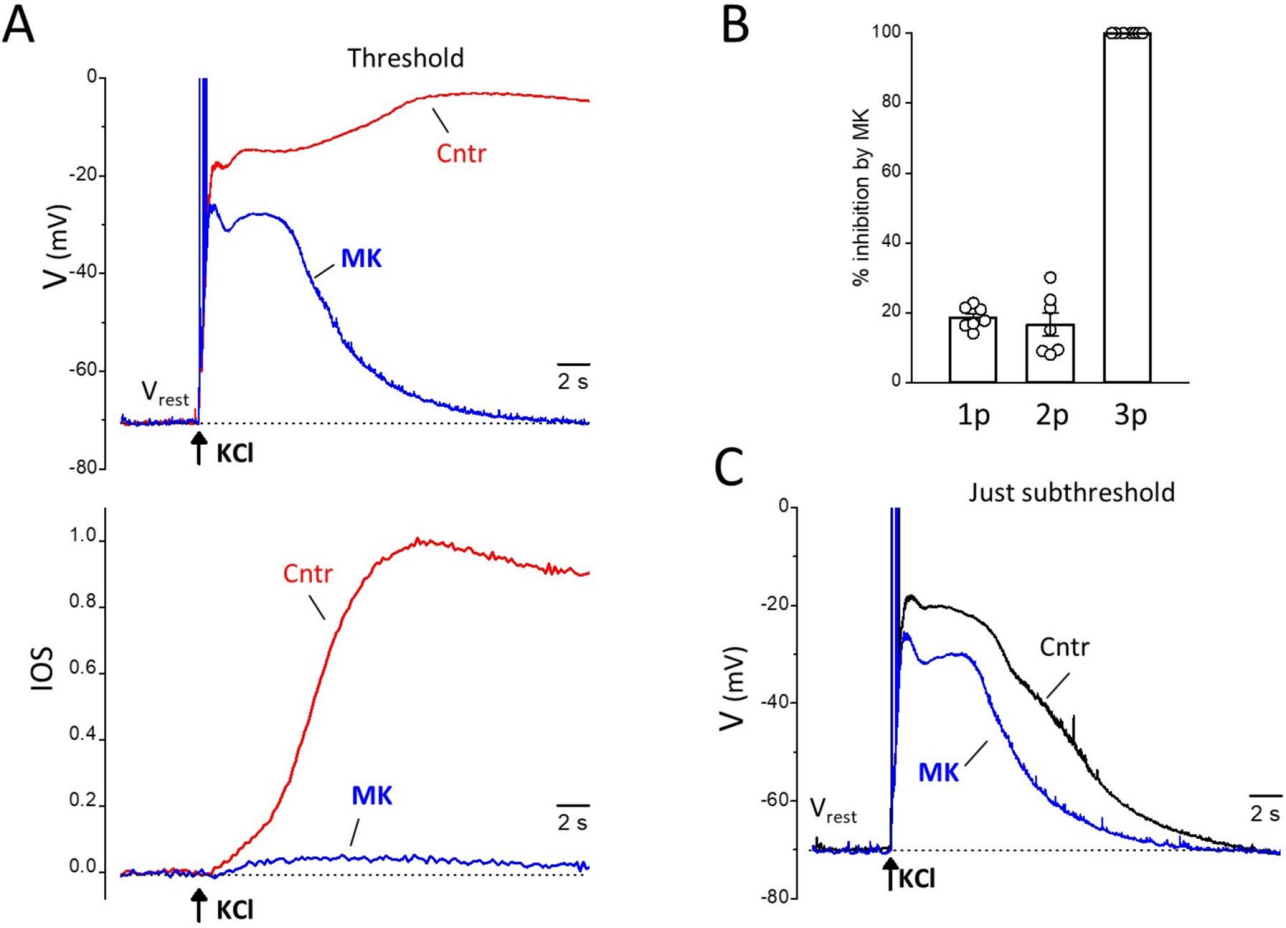
Effect of blocking the NMDARs on the neuronal depolarization elicited near the site of CSD induction by CSD threshold and just subthreshold KCl stimuli. **A** Representative membrane potential traces recorded in response to a CSD threshold KCl stimulus in a L2/3 pyramidal cell located near the KCl puffer (as in Figure 1) in the absence (ctrl, red trace) and presence (MK, blue trace) of MK-801 (50 µM) (upper panel). The corresponding, simultaneously recorded, IOS changes are shown in the lower panel. MK-801 inhibited only 17 % and 24% of the amplitudes (relative to the resting potential) of the 1^st^ and 2^nd^ peak of the depolarization elicited by the CSD threshold stimulus, but completely eliminated the third CSD peak as well as the steep CSD IOS rise. **B** Average percentage of inhibition by MK-801 (20-50 µM) of the peak amplitudes (relative to resting potential) of the depolarizations elicited in L2/3 pyramidal cells located near the KCl puffer by CSD threshold or slightly suprathreshold KCl stimuli (*n* = 7, *N* = 6). 1p, 2p and 3p refer to the 1^st^, 2^nd^ and 3^rd^ (CSD) peaks of the depolarization, as indicated in Figure 1A. **C** Representative membrane potential traces recorded in response to a CSD just subthreshold KCl stimulus (40 ms shorter than the threshold stimulus) in the same L2/3 pyramidal cell of panel A in the absence (ctrl, black trace) and presence (MK, blue trace) of MK-801 (50 µM). MK-801 inhibited 14 % and 19 % of the amplitudes of the 1^st^ and 2^nd^ peak of the depolarization elicited by the just subthreshold stimulus, and completely eliminated the shoulder after the 2nd peak, thus decreasing the duration (at half amplitude) of the just subthreshold depolarization from 10.5 to 7.3 s

The protocol consisted of, first, measuring the CSD threshold in control conditions (by progressively increasing the stimulation intensity as in Fig. 1) and then, after 30 min during which MK-801 was perfused (for 20–25 min), measuring the depolarizations and the IOS changes produced by the control subthreshold and threshold KCl stimuli in the presence of MK-801. With control IOS experiments we established that, after 30 min of sham (extracellular solution) perfusion following the assessment of the control CSD threshold, the CSD threshold was unaltered and the CSD velocity was only slightly reduced (cf. Methods). A saturating concentration of MK-801 (20–50 µM) had a relatively small inhibitory effect on the early depolarization preceding the CSD peak: 19 ± 1% and 17 ± 3% inhibition of the 1st and 2nd peak amplitudes relative to the resting potential, respectively (*n* = 7, *N* = 6, comprising experiments with CSD threshold and slightly suprathreshold KCl stimuli) (Fig. 2A, B). However, MK-801 completely blocked CSD initiation, as shown by the complete elimination of the third depolarization peak and the steep IOS rise (*n* = 7, *N* = 6, Fig. 2A, B). The complete inhibition of CSD initiation by threshold or slightly suprathreshold KCl stimuli after perfusion with MK-801 was observed in additional 11 experiments (*N* = 9) with only IOS recordings. The slow IOS rise preceding CSD initiation was also almost completely inhibited by the NMDAR antagonist.

Considering the depolarization produced by the just subthreshold KCl stimuli (as in the representative experiment in Fig. 2), MK-801 had a relatively small inhibitory effect on the 1st and 2nd peak (15 ± 0.3% and 15 ± 2%, respectively, *n* = 3, *N* = 3, similar to the % inhibition of 1st and 2nd peak amplitudes at the CSD threshold KCl stimulus in the same 3 experiments: 17 ± 2% and 16 ± 5%, respectively; Wilcoxon signed rank test: *p* = 0.4 and 0.8, respectively), but completely eliminated the later shoulder and shortened the duration of the just subthreshold depolarization by 37 ± 5% (Fig. 2C).

**Fig. 3:**
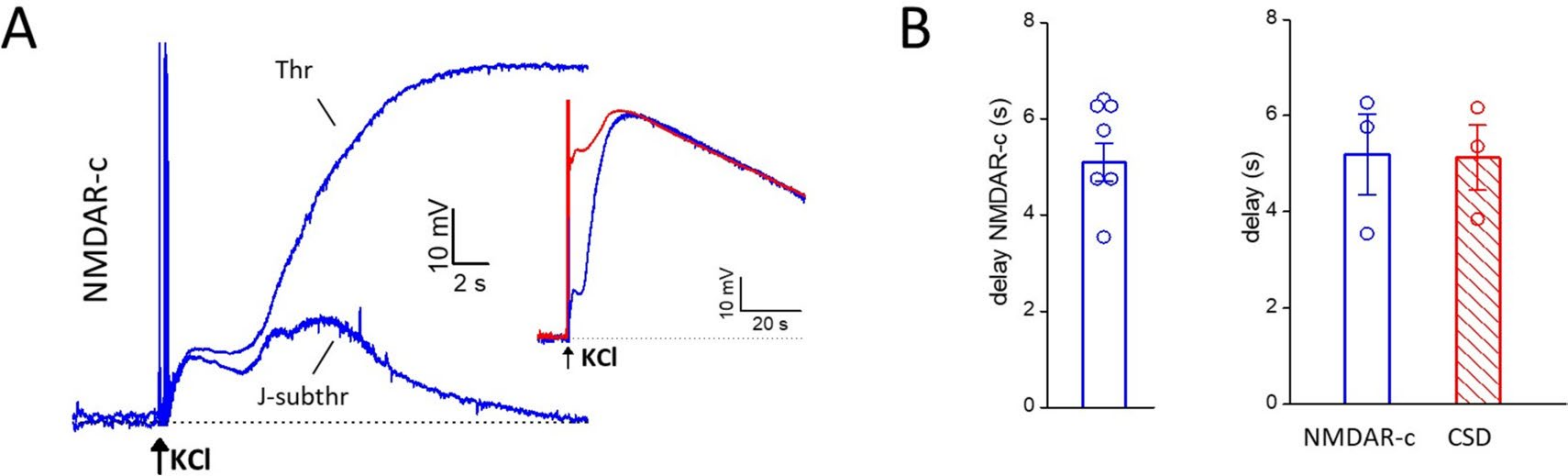
The delayed opening of a sufficient number of NMDARs underlies the ignition of CSD by a threshold stimulus. **A** NMDAR-dependent component (NMDAR-c) of the neuronal depolarizations elicited by the threshold and just subthreshold KCl stimuli, obtained by subtracting the voltages traces recorded after application of MK-801 to the control voltage traces in the absence of drug (same representative experiment as in Figure 2). Inset: entire CSD voltage change (red trace) and NMDAR-dependent component of the depolarization elicited by the threshold stimulus (blue trace) in a compressed time scale. At CSD threshold stimulation, a large delayed NMDAR-dependent component develops with a delay of 5.8 s from the application of KCl, which is similar to the delay of CSD ignition as derived from the steep IOS rise (5.4 s, cf Figure 2A); the inset shows that this component overlaps with and has the same duration of the CSD depolarization (3^rd^ peak, cf Figure 1). At just subthreshold stimulation (J-subthr) a delayed NMDAR-dependent component of much smaller amplitude than at threshold, Thr (10 vs 53 mV) develops with a similar delay (5.4 s). **B** Left: average delay (from the time of the KCl stimulus) of the late NMDAR-c of the depolarization elicited by threshold or slightly suprathreshold KCl stimuli (*n* = 7; *N* = 6). Right: average delay from the time of the KCl stimulus of the late NMDAR-dependent component of the depolarization and of the steep IOS rise typical of CSD in 3 experiments in which voltage and IOS were simultaneously recorded in response to threshold KCl stimuli as in the representative experiment in Figure 2

To display the NMDAR-dependent component (NMDAR-c) of the threshold and just subthreshold depolarizations we subtracted the voltage traces recorded after application of MK-801 to the control voltage traces (Fig. 3A). The difference traces at CSD threshold show a relatively small NMDAR-dependent component of the early depolarization followed by a larger delayed NMDAR-dependent component (Fig. 3A), which developed after a delay of 5.4 ± 0.4 s from the application of the KCl stimulus (*n* = 7, *N* = 6, comprising experiments with CSD threshold and slightly suprathreshold KCl stimuli, Fig. 3B left panel).

In three experiments, in which the delay of CSD ignition in control could be derived from the beginning of the steep IOS rise (including the representative experiment in Fig. 2), the delay with which the late NMDAR-dependent depolarization developed (5.2 ± 0.8 s, *n* = 3, *N* = 3*)* was similar to the delay of CSD ignition (5.1 ± 0.7 s, *n* = 3, *N* = 3; Wilcoxon signed rank test: *p* = 0.8) (Fig. 3B right panel), and the delayed NMDAR-dependent component overlapped with and had the same duration as the CSD depolarization (third peak) (Fig. 3A, inset). The difference voltage traces (control- MK-801) at just subthreshold stimulation show a late NMDAR-dependent component which develops with a similar delay as that at CSD threshold (5.1 ± 0.5 s vs. 5.6 ± 0.4 s, *n* = 3; *N* = 3, Wilcoxon signed rank test: *p* = 0.2) but has a 73 ± 4% smaller amplitude (*n* = 3, *N* = 3) (Fig. 3A). Moreover, in contrast with the prolonged duration of the delayed NMDAR-dependent component at CSD threshold, the late NMDAR-component of the depolarization elicited by just subthreshold stimulation decays to zero relatively rapidly (Fig. 3A). The timing of development of this delayed NMDAR-dependent depolarization suggests that, most likely, it underlies the shoulder (and the prolongation of the depolarization) in the control voltage traces recorded in response to subthreshold KCl stimuli near CSD threshold (Figs. 1A and 2C).

Overall the data suggest that the mechanism underlying the ignition of CSD by a threshold stimulus and not by a just subthreshold stimulus is the opening of a sufficient number of NMDARs (and/or of channels whose opening depends on activation of NMDARs), and that the time necessary to reach this threshold level of NMDAR activation underlies the slow development of the CSD depolarization and its delay relative to the rapid depolarization produced by the threshold KCl stimulus.

**Fig. 4:**
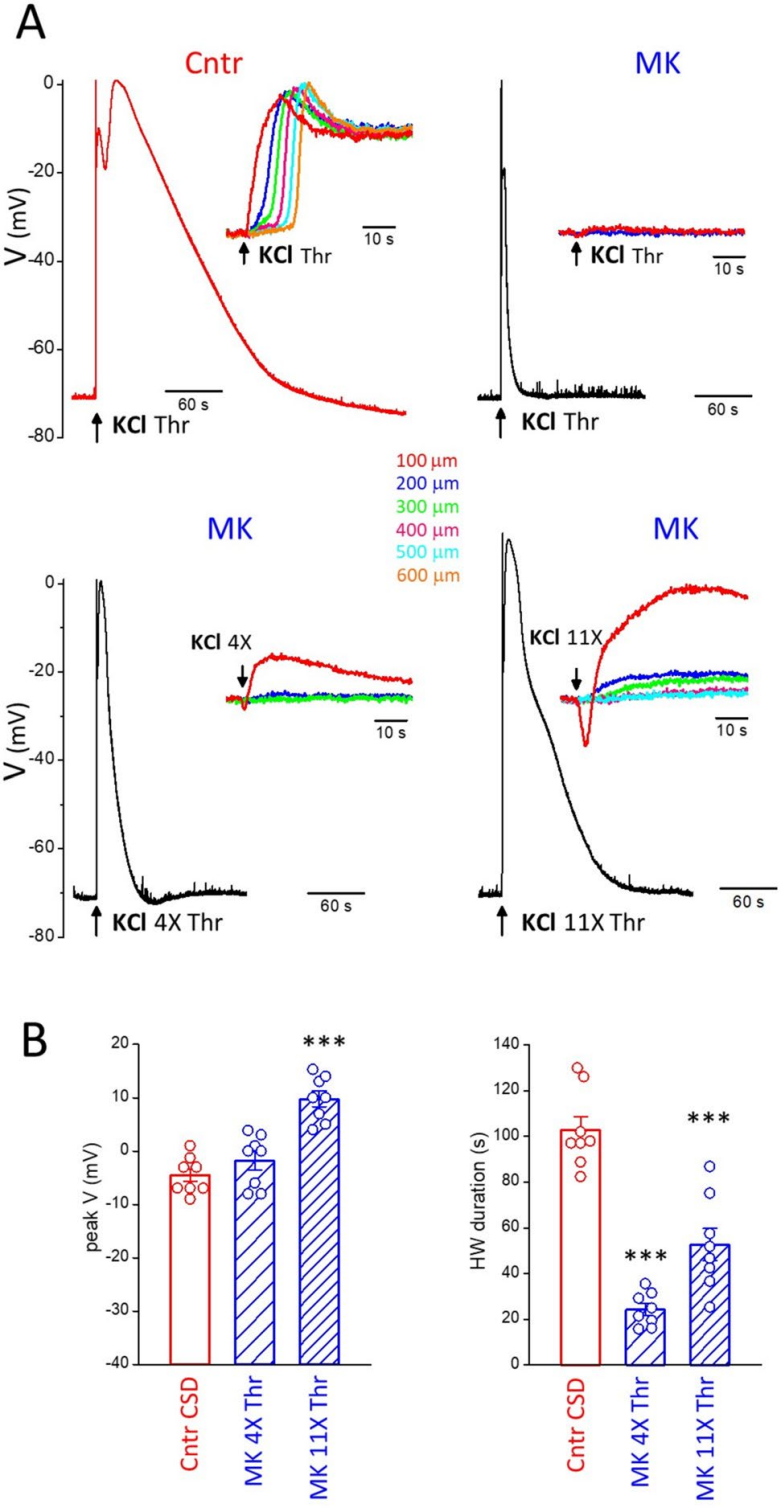
NMDARs are necessary for CSD initiation. **A** Upper panels: representative membrane potential changes recorded in a L2/3 pyramidal cell located near the site of application of KCl in response to a CSD threshold KCl stimulus in control (left) and in the presence of 20 µM MK-801 (right). Lower panels: membrane potential changes recorded in the same L2/3 pyramidal cell in response to largely suprathreshold KCl stimuli of duration 4 times threshold (left) and 11 times threshold (right) in the presence of 20 µM MK-801. The depolarizations produced by these two largely suprathreshold stimuli in the presence of MK-801 (peak values 1 and 10 mV, respectively) are similar or larger than the CSD depolarization (peak value 1 mV), but the durations of the depolarizations are much shorter (16 and 47 s, respectively vs 102 s for the CSD depolarization). The insets in the four panels show the corresponding IOS changes (recorded simultaneously to voltage) at different distances from the KCl puffer. **B** Average peak depolarization voltage (peak V, left panel) and duration of the depolarization at half amplitude (HW duration, right panel) elicited by CSD threshold KCl stimuli in control and by largely suprathreshold KCl stimuli (of average duration 3.8 ± 0.2 and 11.6 ± 0.7 times the CSD threshold duration, *n* = 8, *N* = 6) in the presence of MK-801 (20-50 µM).

The block of CSD ignition by MK-801, when elicited with CSD threshold or slightly suprathreshold KCl stimuli (Fig. 2), leaves open the possibility that largely suprathreshold depolarizing stimuli might be able to ignite CSD even with blocked NMDARs. In other words, does NMDARs block simply increase the threshold for CSD ignition or are NMDARs necessary for CSD ignition? To distinguish between these two possibilities, we increased the KCl stimulus in the presence of MK-801 up to 12 times the control CSD threshold. We found that, after blocking the NMDARs, these largely suprathreshold stimuli did not induce CSD, despite the fact that they depolarized the neuronal membrane even more than the control CSD (Fig. 4). In the presence of MK-801, stimuli 4 and 12 times larger than the control CSD threshold depolarized the membrane to -2 ± 2 mV and 10 ± 1 mV, respectively (in 8 experiments in which the peak CSD depolarization was − 5 ± 1 mV), but the depolarizations were shorter than the CSD (HW duration: 24 ± 3 and 53 ± 7 s, respectively, vs. 103 ± 6 s for CSD, *n* = 8, *N* = 6; paired t-test: *p* = 2.7 × 10^− 6^ and 1.8 × 10^− 4^, respectively) (Fig. 4) and, most importantly, did not propagate, as clearly shown by the IOS changes at different distances from the KCl stimulus (Fig. 4A, insets). So, it is not the level of extracellular K^+^ and of the neuronal depolarization per se that determines the initiation of CSD, which rather requires depolarization (and/or K^+^)-dependent processes which develop relatively slowly and involve the necessary opening of a sufficient number of NMDARs.

AMPA/kainate glutamate receptors do not significantly contribute, since the early depolarization preceding CSD and the subthreshold depolarizations were barely affected by 50 µM NBQX (not shown). Accordingly, blocking AMPA/kainate glutamate receptors did not affect CSD threshold and velocity, as shown by the unaltered CSD threshold measured after perfusion with NBQX (CSD threshold: 277 ± 25 ms in NBQX vs. 257 ± 22 ms in control, *n* = 6, *N* = 4, Wilcoxon signed rank test: *p* = 0.15), and the slight, hardly significant, decrease in CSD velocity, similar to that measured after sham perfusion (1.80 ± 0.09 mm/min in NBQX vs. 2.18 ± 0.2 in control, *n* = 6, *N* = 4, paired t-test, *p* = 0.04).

**Fig. 5:**
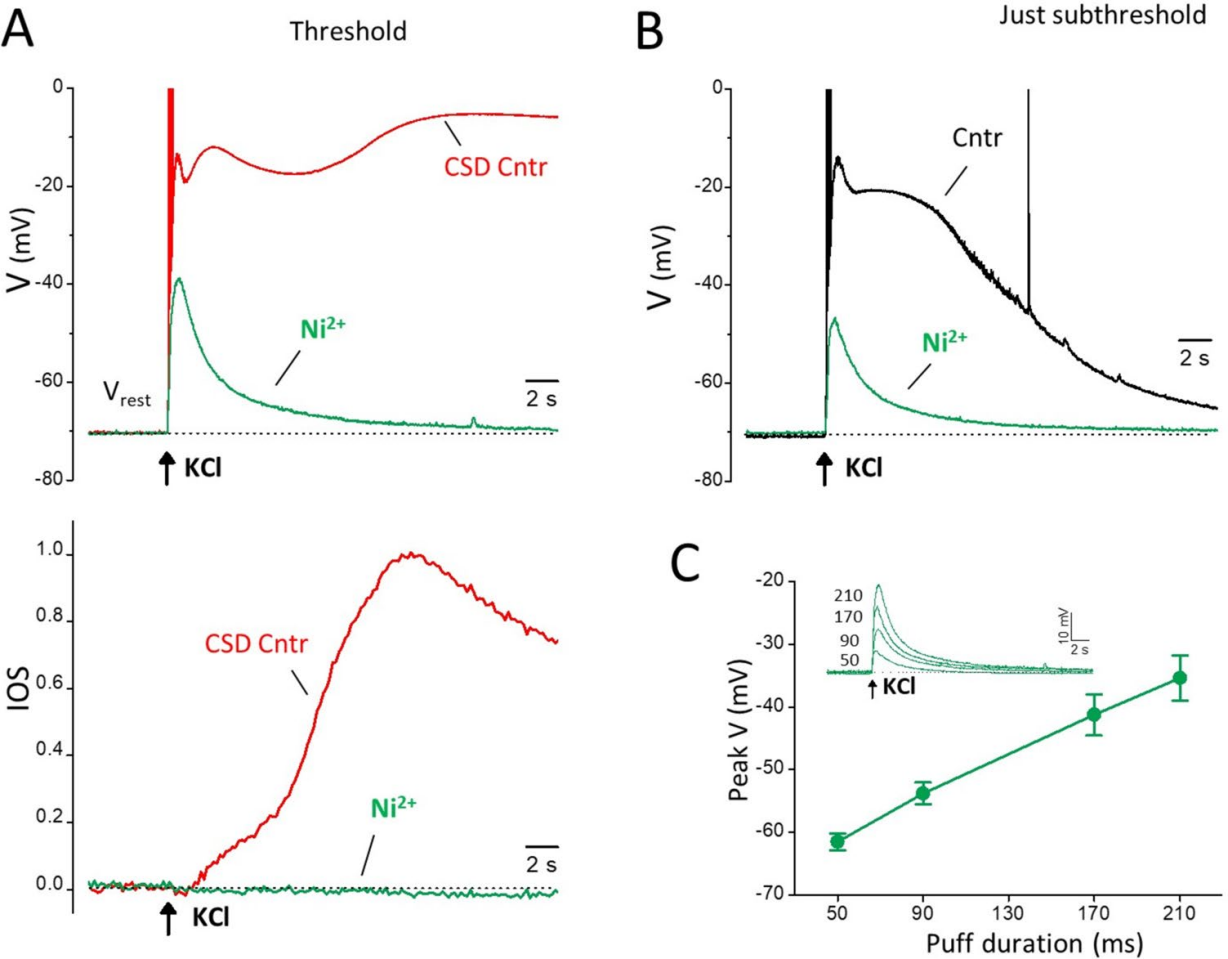
Effect of blocking the voltage-gated Ca^2+^ channels on the neuronal depolarization elicited near the site of CSD induction by CSD threshold and just subthreshold KCl stimuli. **A** Representative membrane potential traces recorded in response to a CSD threshold KCl stimulus in a L2/3 pyramidal cell located near the KCl puffer (as in Figure 1) in the absence (ctrl, red trace) and presence (Ni^2+^, green trace) of a saturating concentration of Ni^2+^ (5 mM) (upper panel). The corresponding, simultaneously recorded, IOS changes are shown in the lower panel. Ni^2+^ completely inhibited the CSD peak depolarization (3^rd^ peak) as well as the steep CSD IOS rise. Ni^2+^ also eliminated the 2^nd^ peak of the early depolarization as well as the slow IOS rise preceding CSD initiation, and it inhibited 45 % of the amplitude (relative to the resting potential) of the 1^st^ peak of the depolarization elicited by the CSD threshold stimulus. **B** Representative membrane potential traces recorded in response to a CSD just subthreshold KCl stimulus in the same L2/3 pyramidal cell of panel A in the absence (ctrl, black trace) and presence (Ni^2+^, green trace) of Ni^2+^. Ni^2+^ inhibited 57 % of the amplitude (relative to the resting potential) of the 1^st^ peak of the depolarization elicited by the just subthreshold stimulus, and eliminated both the 2^nd^ peak and its shoulder, thus decreasing the duration (at half amplitude) of the just subthreshold depolarization from 11.7 to 2.1 s. **C** Average peak voltage of the depolarizations produced by KCl stimuli of increasing intensity in the presence of Ni^2+^ (5 mM) as a function of stimulus intensity (KCl puff duration) (*n*= 4; *N* =4). Inset: representative membrane potential traces recorded in response to subthreshold KCl stimuli of increasing intensity up to the threshold stimulus in the presence of Ni^2+^ (5 mM) in the same L2/3 pyramidal cell of panels A, B

We investigated the role of voltage-gated calcium channels in the early threshold and subthreshold depolarizations and in CSD ignition by measuring the effect of Ni^2+^ (a blocker of voltage-gated calcium channels, Ca_V_) using the same protocol employed to test the effect of MK-801 and NBQX. Ni^2+^, at a concentration (5 mM) that blocks all the different types of Ca_V_ channels [29], completely blocked CSD initiation by a threshold KCl stimulation, as shown by the complete elimination of the third depolarization peak and the steep IOS rise (Fig. 5A, representative of 5 experiments with both IOS and V recordings, *N* = 5; the complete inhibition of CSD ignition by Ni^2+^ was observed in additional 6 experiments with only IOS recording, *N* = 6). Ni^2+^ also had a strong inhibitory effect on the early depolarization and eliminated its second peak, as well as the slow IOS rise preceding CSD initiation (Fig. 5A). Likewise, considering the depolarization produced by the just subthreshold KCl puff, Ni^2+^ eliminated both its second peak and the NMDAR-dependent (cf. Figures 2 and 3) shoulder (Fig. 5B), and it shortened the duration of the just subthreshold depolarization by 78 ± 4% (*n* = 4, *N* = 4). Likely, the depolarizations measured in the presence of Ni^2+^, which depend on the intensity of the KCl stimulus as shown in Fig. 5C, reflect the direct depolarizing effect of KCl on membrane potential. The opening of Ca_V_ channels directly and/or indirectly contributes to the first peak and is (directly and/or indirectly) responsible for the second peak of both the subthreshold depolarizations and the early threshold depolarization. Blocking the Ca_V_ channels prevents the delayed opening of the NMDARs responsible for the shoulder/prolongation of the subthreshold depolarizations and for the ignition of CSD above a certain level.

**Fig. 6:**
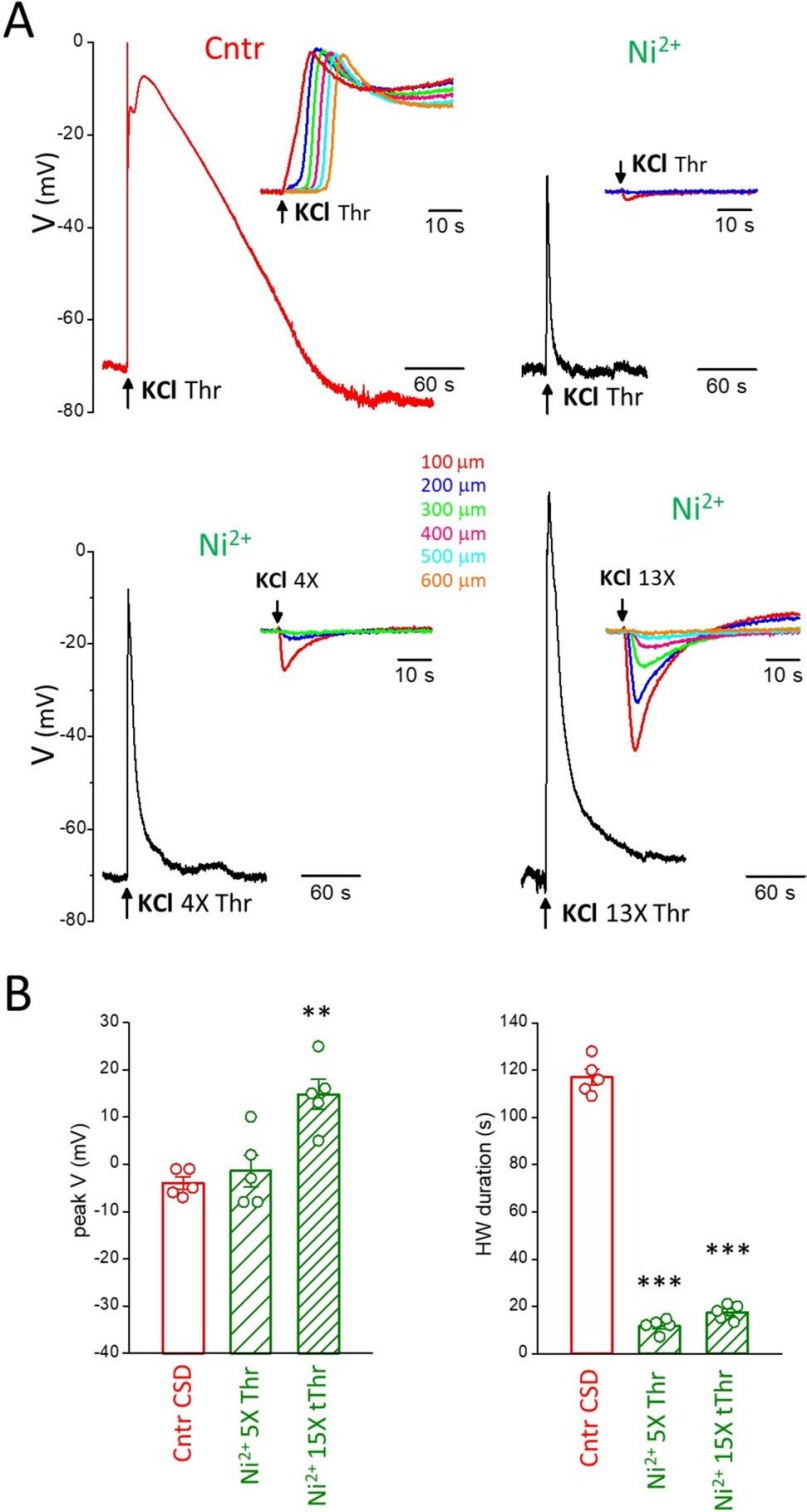
Voltage-gated Ca^2+^ channels are necessary for CSD initiation. **A** Upper panels: representative membrane potential changes recorded in a L2/3 pyramidal cell located near the site of application of KCl in response to a CSD threshold KCl stimulus in control (left) and in the presence of 5 mM Ni^2+^ (right). Lower panels: membrane potential changes recorded in the same L2/3 pyramidal cell in response to largely suprathreshold KCl stimuli of duration 4 times threshold (left) and 13 times threshold (right) in the presence of 5 mM Ni^2+^. The depolarizations produced by these two largely suprathreshold stimuli in the presence of Ni^2+^ (peak values -8 and 13 mV, respectively) are similar or larger than the CSD depolarization (peak value -7 mV), but the durations of the depolarizations are much shorter (7 and 15 s, respectively vs 112 s for the CSD depolarization). The insets in the four panels show the corresponding IOS changes (recorded simultaneously to voltage) at different distances from the KCl puffer. **B** Average peak depolarization voltage (peak V, left panel) and duration of the depolarization at half amplitude (HW duration, right panel) elicited by CSD threshold KCl stimuli in control and by largely suprathreshold KCl stimuli (of average duration 5.0 ± 0.3 and 14.8 ± 0.8 times the CSD threshold duration, *n*= 5, *N* = 5) in the presence of Ni^2+^ (5 mM)

The block of CSD ignition by Ni^2+^ at CSD threshold KCl stimuli shown in Fig. 5 leaves open the possibility that largely suprathreshold depolarizing stimuli might be able to ignite CSD even with blocked Ca_V_ channels. In other words, does Ca_V_ channel block simply increases the threshold for CSD ignition or are Ca_V_ channels necessary for CSD ignition? To distinguish between these two possibilities, we increased the KCl stimulus in the presence of Ni^2+^ up to 15 times the control CSD threshold. After blocking the Ca_V_ channels, CSD was not induced by these largely suprathreshold stimuli, despite the fact that the neuronal membrane was depolarized even more than during the control CSD (Fig. 6).

In the presence of Ni^2+^, stimuli 5 and 15 times larger than the control CSD threshold depolarized the membrane to -1 ± 3 mV and 15 ± 3 mV, respectively (in 5 experiments in which the CSD depolarization was − 4 ± 1 mV), but the depolarizations were much shorter than the CSD (HW duration: 12 ± 1 s and 17 ± 1 s, respectively, vs. 117 ± 3 s for CSD, *n* = 5, *N* = 5, paired t-test, *p* = 4 × 10^− 6^ and 6 × 10^− 6^, respectively) (Fig. 6) and, most importantly, did not propagate, as clearly shown by the IOS changes at different distances from the KCl stimulus (Fig. 6A, insets). This confirms that it is not the level of extracellular K^+^ or of neuronal depolarization per se that determines the initiation of CSD, which rather requires the necessary opening of Ca_V_ channels and of a sufficient number of NMDARs.

**Fig. 7:**
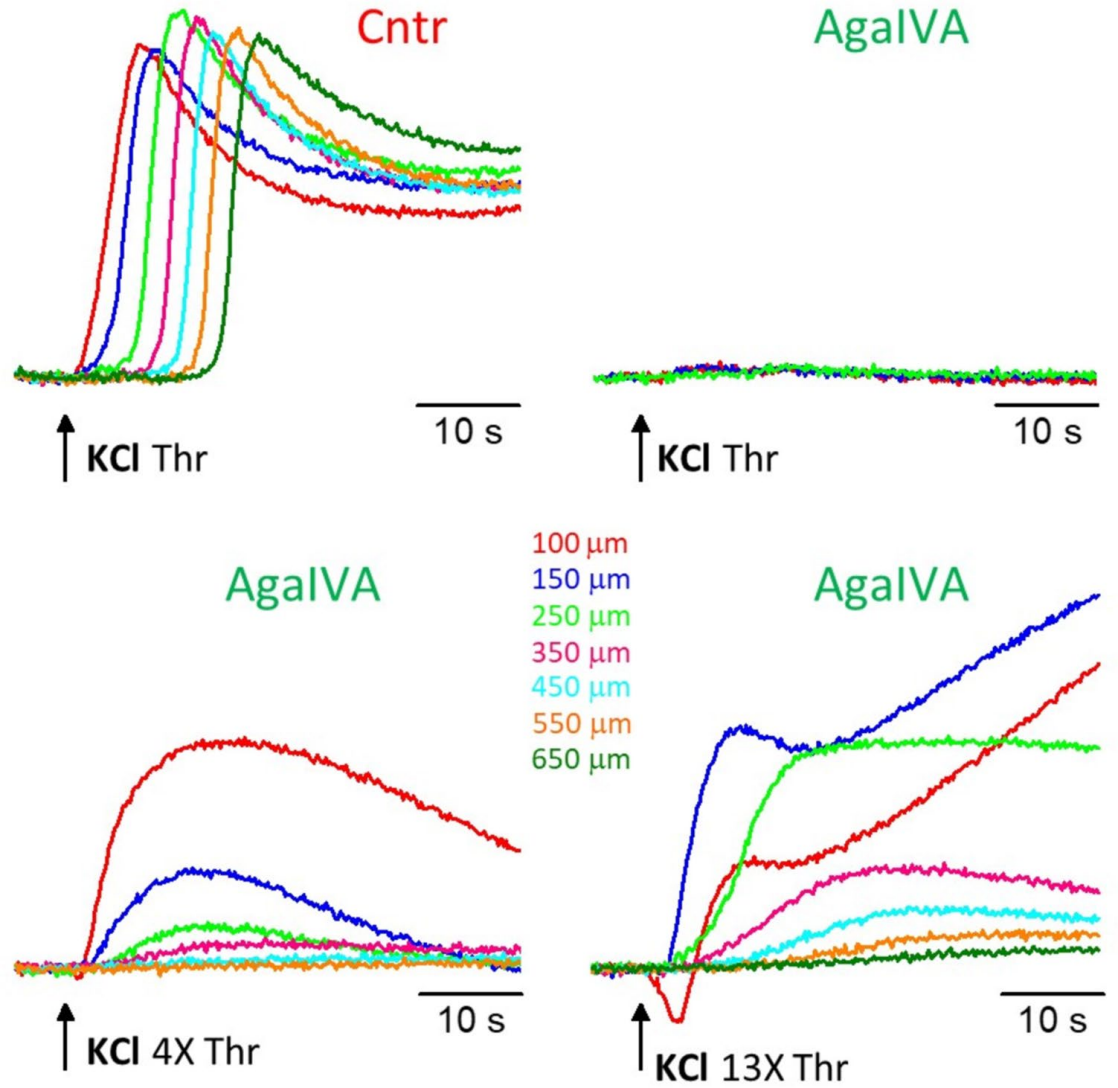
Blocking Ca_V_2.1 Ca^2+^ channels prevents CSD initiation by suprathreshold stimuli at least four times larger than the control CSD threshold. Upper panels: representative IOS changes recorded at different distances from the site of KCl application (cf inset in Figure 1A) in response to a CSD threshold KCl stimulus in control (left) and in the presence of 400 nM ω-AgaIVA (right). Lower panels: IOS changes recorded in the same slice at the same distances in response to largely suprathreshold KCl stimuli of duration 4 times threshold (left) and 13 times threshold (right) in the presence of 400 nM ω-AgaIVA. After blocking Ca_V_2.1 channels, almost no IOS changes were recorded in response to threshold stimulation and only slow non-propagating IOS changes whose amplitude rapidly declined with distance from the site of KCl application were recorded in response to a stimulus 4 times larger than the threshold stimulation. The steeper and biphasic IOS change recorded at 150 µm in response to a KCl stimulus 13 times threshold and the corresponding IOS change recorded at 100 um (which is distorted by the large artefactual inward IOS change produced by the very high KCl concentration) can be interpreted as indicative of a CSD-like event propagating for a short distance (up to 250 µm) from the site of KCl application. Farther than this distance, the amplitudes of the IOS changes rapidly declined

 Given the dominant role played by Ca_V_2.1 channels in controlling glutamate release at cortical synapses [18, 20] and the fact that gain-of-function mutations in Ca_V_2.1 channels cause FHM1 and, by increasing glutamate release, facilitate experimental CSD [23, 30, 31], we asked whether Ca_V_2.1 channels are necessary for CSD initiation in wild-type mice. To answer this question, we measured the IOS changes produced by CSD threshold and largely suprathreshold KCl stimuli at different distances from the site of KCl application in the presence of a saturating concentration (400 nM) of ω-AgaIVA, a specific blocker of Ca_V_2.1 (P/Q-type calcium) channels [29]. After blocking Ca_V_2.1 channels, CSD was not induced by KCl stimuli up to 4 times larger than the control CSD threshold, as shown by the slow changes in IOS, which did not propagate and whose amplitude rapidly declined with distance (Fig. 7, representative of *n* = 9 experiments, *N* = 7). Stimuli 12 times larger than the average control CSD threshold elicited IOS changes indicative of a CSD-like event at the site of KCl application, which propagated for a short distance (up to 250 μm in the representative experiment in Fig. 7); further than this distance, the amplitudes of the IOS changes rapidly declined. We can conclude that Ca_V_2.1 channels are fundamental for CSD initiation (since their block increases several folds, ≥ 4 times, the CSD threshold at the site of application of the KCl stimuli), and are necessary for CSD propagation (since the “aborted CSD” observed with very large suprathreshold stimuli, 13 times the control CSD threshold, did not propagate beyond 294 ± 34 μm from the site of KCl application, *n* = 9, *N* = 7). The fact that blocking all the different types of Ca_V_s totally prevented CSD initiation by suprathreshold stimuli up to 15 times larger than the control CSD threshold is consistent with the evidence that also Ca_V_2.2 and Ca_V_2.3 channels are involved in CSD initiation, although with a minor role compared to Ca_V_2.1 channels [16].

## Discussion

Our study provides novel insights into the ion channels that are necessary for CSD initiation and into the mechanisms underlying the initiation of CSD by a threshold focal KCl stimulus and the failure to initiate it by a just subthreshold stimulus. They can be summarized as follows.

The onset of CSD does not coincide with the rapid strong depolarization produced by a threshold KCl puff on neurons located close to the puffer, but is delayed by several seconds (average 7.4 ± 0.5 s). The mechanism underlying the ignition of CSD by a threshold stimulus and not by a just subthreshold stimulus is the opening of a threshold level of NMDARs (and/or of channels whose opening depends on sufficient activation of NMDARs). The NMDARs (and/or the NMDAR-dependent channels), essential for CSD initiation at threshold, open with a delay of several seconds after the KCl puff, which is similar to the delay of CSD initiation. Thus, the time necessary to reach the threshold level of NMDAR activation underlies the delay of CSD initiation relative to the rapid depolarization produced by the KCl stimulus. Interestingly, beginning at a time before CSD onset similar to this delay, a slow rise of extracellular glutamate and an increase in glutamatergic plumes frequency was observed at the site of CSD induction by application of a threshold KCl concentration in vivo, in awake head-fixed mice [10]. This suggests that the time necessary to reach the level of NMDAR activation which is critical for CSD initiation may correspond to the time necessary to increase the plumes of glutamate and/or the extracellular glutamate above a critical threshold level [10]. Since the plumes of glutamate may be considered a marker of inefficient glutamate clearance [10], one can hypothesize that a critical level of impairment of glutamate clearance (due to decreased cycling rate of glutamate transporters consequent to the depolarization and the extracellular K^+^ increase and the intracellular accumulation of Na^+^ and glutamate produced by the KCl stimulus) may be necessary to rise glutamate above the critical level which leads to delayed cooperative activation of synaptic and extrasynaptic NMDARs [32] above the threshold level indispensable to initiate CSD.

The changes in amplitude and shape of the depolarizations produced by increasing subthreshold stimulations (characterized by the appearance, above a certain stimulus intensity, of a second depolarization peak of increasing amplitude following the initial rapid depolarization and by a later shoulder of increasing amplitude) suggest that the sequential opening of different cationic channels with different time courses and different dependence on the K^+^ stimulus occurs. Only a relatively small fraction of the first and second peaks of the subthreshold depolarizations and of the early threshold depolarization preceding CSD initiation is due to the rapid opening of NMDARs. The delayed opening of additional NMDARs underlies the shoulder and the prolongation of the depolarization at relatively high subthreshold stimuli, and the level of activation of these NMDARs is critical for making the depolarization regenerative and initiating CSD at threshold stimulation.

The opening of voltage-gated calcium channels directly (and/or indirectly) underlies the second peak of the subthreshold depolarizations and of the early threshold depolarization preceding CSD initiation, and is necessary for the delayed opening of the NMDARs underlying the shoulder and the prolongation of the depolarization at relatively high subthreshold stimuli. CSD initiation at threshold stimulation is prevented by inhibition of Ca_V_ channels with Ni^2+^ and also by specific inhibition (with ω-AgaIVA) of the Ca_V_2.1 channels, which play a dominant role in controlling glutamate release at cortical synapses [18, 20]. Most likely, the block of CSD initiation is due to the inhibition of the presynaptic Ca_V_ (in particular Ca_V_2.1) channels and the consequent inhibition of glutamate release evoked by the KCl stimulus (through both action potentials generation and direct depolarization of presynaptic terminals). Possibly, also the inhibition of the release of other neurotransmitters (e.g. ATP) might contribute, if this induces a delayed release of glutamate from other cells (astrocytes and/or microglia) which might contribute to the delayed activation of NMDARs critical for CSD initiation.

The kinetics of development of the Ca_V_-dependent second peak of the subthreshold depolarizations and of the early depolarization preceding CSD initiation suggest that, likely, it does not result from the direct opening of postsynaptic Ca_V_ channels (which rather may directly contribute to the first peak), but it is due to activation of other postsynaptic cationic channels whose opening depends on and temporally follows the opening of Ca_V_ channels. The nature of these cationic channels and the role they may possibly play in CSD initiation remain unclear. Also unclear is whether the Ca_V_ channels from which they depend are postsynaptic, as e.g. could be the case if the cationic channels are [Ca^2+^]_in_-dependent and their opening occurs above a certain [Ca^2+^]_in_, or presynaptic, as e.g. could be the case if the opening of the cationic channels depends on sufficient Ca_V_-dependent neurotransmitter release. The lack of effect of NBQX and the little effect of MK-801 on the second peak depolarization exclude that these cationic channels are glutamate receptors.

Our study provides novel insights into the ion channels that are ***necessary*** for initiation of CSD. The finding that blocking either NMDARs or Ca_V_ channels prevents initiation of CSD by even very intense, largely suprathreshold stimuli (up to 12–15 times the threshold stimulation) shows that both Ca_V_ channels and NMDARs are necessary for initiation of CSD (by focal high KCl stimulation). Near the site of application of the suprathreshold stimuli, neurons are completely depolarized but, in the presence of MK-801 or Ni^2+^, the depolarization (which is shorter than the typical CSD depolarization) does not propagate (and hence is not a CSD). This provides strong evidence that it is not the level of extracellular K^+^ and of the neuronal depolarization per se that determines the initiation of CSD. Consistent with and supporting this conclusion is also the finding that the early depolarization produced by threshold stimulation was quite different in different experiments and was similar to that produced by a just subthreshold stimulation.

Based on the finding that the early depolarization recorded in pyramidal cells located far (> 200 μm) from the KCl puffer was not affected by the selective block of NMDARs in the recorded individual neurons (by either intracellular MK-801 or genetic ablation of GluN1), Mei et al. [12] concluded that NMDARs are neither necessary nor involved in CSD initiation by KCl pulses in hippocampal slices. There may be several possible explanations for the apparently conflicting findings. Mei et al. [12] did not investigate CSD at the initiation site and therefore their findings concern the mechanism of CSD propagation (i.e. initiation of CSD in contiguous tissue) rather than the mechanism of CSD initiation at the induction site. The findings of Mei et al. [12] are quite similar to those reported in hippocampal slices by Aiba and Shuttleworth [33], who showed that bath application of a low concentration of D-AP5 did not affect the early depolarization of pyramidal cells (located far from the site of focal KCl application) and did not affect the initiation of the CSD depolarization at the distant site but reduced the duration of the CSD depolarization (as in [12]). However, higher concentrations of D-AP5 did block CSD measured far from the initiation site [34], indicating that CSD propagation and/or initiation are less sensitive to inhibition by a NMDAR antagonist than the duration of the CSD depolarization (or different NMDARs subtypes are involved in sustained depolarization and in CSD initiation/propagation) [3]. This suggests that inhibition of NMDARs by intracellular MK-801 or genetic ablation of GluN1 (in particular NMDARs in the apical dendrites, which are the first to be depolarized during CSD propagation [3, 33, 35, 36] may have been incomplete in [12]. Possibly, regional (cerebral cortex vs. hippocampus) differences in CSD mechanisms [3] might also contribute to the apparently conflicting findings.

How general are our conclusions regarding the CSD initiation mechanisms if we consider other methods of CSD induction in normally metabolizing brain tissue? They appear valid for optogenetic CSD induced by focal light stimulation to depolarize pyramidal cells expressing channelorodopsin2 in anesthetized [37] or awake head-fixed mice [38]. MK-801 blocked the initiation of optogenetic CSD at the illumination site by light stimuli near threshold [38] and three times threshold [37], without affecting the light-induced field potentials recorded at the illumination site. Interestingly, in [37] the optogenetic CSD started with a delay of up to 10–20 s after the end of the illumination (and the associated negative field potential) and the delay was independent of the potential shift amplitude. During the post-illumination, pre-CSD phase, light-induced field potentials and local [K]_e_ diminished and in some cases completely returned to baseline by the time CSD developed.

Our conclusions appear also in large part valid for CSD initiation by veratridine in awake head-fixed mice [10]. CSD initiation was preceded by slow rises of extracellular glutamate and plumes frequency at the initiation site [10]. The levels of both glutamate and glutamatergic plumes frequency just prior to CSD onset by a threshold veratridine stimulation were quite similar in wild-type and familial hemiplegic migraine type 2 knockin mice (FHM2 mice, carrying a loss-of-function mutation in the astrocytic Na^+^/K^+^ ATPase [39]), despite the fact that the threshold concentration of veratridine was lower in FHM2 mice [10], in agreement with their lower threshold for CSD induction using focal KCl or focal electrical stimulation [27, 32, 39]. This evidence of a threshold level of extracellular glutamate necessary for CSD initiation, regardless of genotype, is consistent with and supports our conclusion of a threshold level of NMDAR activation necessary for CSD initiation. Moreover, the finding that the rise of extracellular glutamate to the threshold level necessary for CSD initiation was faster in FHM2 mice (with reduced rate of glutamate clearance at cortical synapses [27]) compared to wild-type mice may be consistent with and support the hypothesis that the delay of CSD initiation reflects the time necessary to reach the threshold levels of glutamate and NMDAR activation consequent to impairment of glutamate clearance. Interestingly, Ni^2+^ completely inhibited the glutamate rise preceding CSD initiation by veratridine in FHM2 mice, thus showing its dependence on Ca_V_-initiated release, and prevented CSD initiation by threshold stimulation in most FHM2 mice [10]. However, in most mice, Ni^2+^ did not prevent CSD initiation by suprathreshold stimulation, suggesting that, in contrast with KCl-induced CSD, Ca_V_ channels and Ca_V_-dependent glutamate release are not necessary for initiation of CSD by high concentrations of veratridine, which likely activates other processes contributing to CSD ignition [10].

Our conclusions do not appear valid for CSD induced in cortical slices by focal light stimulation to depolarize GABAergic interneurons expressing channelorhodopsin2 [40]. Ca_V_ channels and NMDARs do not appear necessary for initiation of this cerebral cortex-specific optogenetic CSD, since initiation of CSD induced by likely suprathreshold light stimuli was not prevented by the block of Ca_V_ channels or NMDARs (although the incomplete inhibition of CSD propagation might suggest incomplete block, in particular of NMDARs given the relatively low concentration of antagonist). However, the inhibition of Ca_V_ channels or NMDARs delayed CSD initiation, suggesting that both Ca_V_ channels and NMDARs likely contribute to the initiation of CSD (and it remains to be seen whether their inhibition would prevent it at threshold stimulation).

Overall, the data on the mechanisms underlying the initiation of CSD by a threshold focal stimulus (KCl, veratridine, optogenetic depolarization of pyramidal cells) in wild-type mice as well as the data on the mechanisms underlying the facilitation of CSD initiation in FHM1 and FHM2 knockin mice [10, 23, 27, 32] support the following model of CSD initiation in normally metabolizing brain tissue. CSD initiation requires Ca_V_ channels activation and Ca_V_-dependent glutamate (and possibly other neurotransmitters) release as well as impaired handling of glutamate release, which result in threshold levels of extracellular glutamate and glutamatergic plumes and cooperative activation of (synaptic and extrasynaptic) NMDARs on the apical dendrites of pyramidal cells above a threshold level. Increased susceptibility to CSD in both FHM1 and FHM2 knockin mice is due to the fact that these threshold levels are reached with stimuli of lower intensity.

Shedding light on the CSD initiation process in normally metabolizing brain tissue, our data give insights into potential mechanisms of CSD initiation in the brain of migraineurs. In particular, they suggest that initiation of a “spontaneous” CSD may be favored by conditions leading to excessive activation (above the critical threshold level) of synaptic and extrasynaptic NMDARs in the apical dendrites of cortical pyramidal cells, due to excessive synaptic excitation and local elevation of glutamate above a critical threshold level. This may occur as a consequence of an imbalance between Ca_V_-dependent glutamate release and clearance of glutamate at cortical excitatory synapses, which might be created by enhanced Ca_V_2.1-dependent glutamate release (as in FHM1) [23, 26, 41] or reduced clearance of glutamate by astrocytes (as in FHM2) [10, 27] or other mechanisms, which remain to be uncovered (as in common migraine with aura). Besides excessive synaptic excitation, activation of dendritic synaptic and extrasynaptic NMDARs above the critical threshold level likely requires disinhibition of the dendrites [26, 42]. This might occur in certain conditions as a consequence of dysfunctional regulation of the excitatory-inhibitory balance in specific cortical (micro)circuits [43].

Peri-infarct and post-trauma recurrent spreading depolarizations are frequently observed in infarct and brain trauma, and they affect the disease outcome [44–47]. According to preclinical studies, the mechanisms of initiation of spreading depolarizations in metabolically impaired brain tissue are different from those of CSD initiation in normally metabolizing tissue and involve other ion channels, besides Ca_V_s and NMDARs both of which do not seem necessary for initiation of spreading depolarizations [3]. Nonetheless, our findings might be relevant for the outcome of neurological diseases such as infarct or brain trauma. For example, they may help to explain enhanced vulnerability to stroke and worse stroke outcome as well as increased brain edema formation and worse outcome following head injury in FHM1 knockin mice, and possibly, patients [48–50]. In fact, the worse stroke and brain trauma outcomes in FHM1 knockin compared to wild-type mice correlate with the increased frequency of peri-infarct and post-brain trauma spreading depolarizations measured in these mutants [48, 50]. Interestingly, the differences in infarct volume and neurological symptoms between FHM1 and wild-type mice were abolished by pre-ischemic treatment with MK801 [48]. This is consistent with both enhanced Ca_V_-dependent NMDAR activation (due to enhanced glutamate release and, possibly, impaired handling of it) as a mechanism for the increased frequency of peri-infarct spreading depolarizations in the FHM1 mice and with prolonged activation of NMDARs and calcium influx during the spreading depolarizations as cellular mechanisms of neuronal injury [33].

## Conclusions

Our investigation of the mechanisms underlying CSD initiation by focal application of high KCl in acute cerebral cortex slices shows that both NMDARs and Ca_V_ channels are necessary for CSD initiation, which is not determined by the level of extracellular K^+^ or neuronal depolarization per se, but requires the Ca_V_-dependent activation of a threshold level of NMDARs. This occurs with a delay of several seconds relative to the rapid depolarization produced by the KCl stimulus. We hypothesize that this delay might reflect the time necessary to reach threshold levels of extracellular glutamate-glutamatergic plumes consequent to impairment of glutamate clearance. Our data give insights into potential mechanisms of initiation of “spontaneous” CSDs in the brain and hence into the unknown mechanisms which make the brain of migraineurs susceptible to CSD, the neurophysiological correlate of migraine aura and a trigger of the migraine pain mechanisms.

## Data Availability

Most of the data generated or analysed during this study are included in this published article and all data are available from the corresponding author on reasonable request.

## Abbreviations

CSD: Cortical spreading depression
NMDARs: Glutamate NMDA receptors
Ca_V_ channels: Voltage-gated Ca^2+^ channels
FHM1: Familial hemiplegic migraine type 1
IOS: Intrinsic optic signal
L2/3: Layer 2/3
HW duration: Duration at half amplitude
FHM2: Familial hemiplegic migraine type 2
