# Correction: Mechanisms of initiation of cortical spreading depression

**DOI:** 10.1186/s10194-023-01702-1

**Published:** 2023-12-20

**Authors:** Marina Vitale, Angelita Tottene, Maral Zarin Zadeh, K. C. Brennan, Daniela Pietrobon

**Affiliations:** 1 Department of Biomedical Sciences, University of Padova, 35131 Padua, Italy; 2 Department of Neurology, University of Utah School of Medicine, Salt Lake City, UT 84108 USA; 3 Padova Neuroscience Center (PNC), University of Padova, 35131 Padua, Italy

**Correction: J Headache Pain 24, 105 (2023)**

**https://doi.org/10.1186/s10194-023-01643-9**

In the original version of this article [1], on page 2, in the second paragraph of the right-hand column, the text reads: “ On the other hand, mutant mice carrying mutations, which cause partial loss-of-function of CaV2.1 channels and reduced K^+^-evoked glutamate rise, showed a lower CSD stimulation threshold” but it should have read: “ On the other hand, mutant mice carrying mutations, which cause partial loss-of-function of CaV2.1 channels and reduced K + -evoked glutamate rise, showed **an increased** CSD stimulation threshold”.

The original article has been corrected.
